# Spatio-temporal modelling of weekly malaria incidence in children under 5 for early epidemic detection in Mozambique

**DOI:** 10.1038/s41598-018-27537-4

**Published:** 2018-06-18

**Authors:** Kathryn L. Colborn, Emanuele Giorgi, Andrew J. Monaghan, Eduardo Gudo, Baltazar Candrinho, Tatiana J. Marrufo, James M. Colborn

**Affiliations:** 1 Department of Biostatistics and Informatics, University of Colorado Anschutz Medical Campus, Aurora, CO USA; 2 Lancaster Medical School, Lancaster University, Lancaster, UK; 3 National Center for Atmospheric Research, Boulder, CO USA; 4 Instituto Nacional de Saude, Maputo, Mozambique; 5 National Malaria Control Program, Maputo, Mozambique; 6 Clinton Health Access Initiative, Boston, MA USA

## Abstract

Malaria is a major cause of morbidity and mortality in Mozambique. We present a malaria early warning system (MEWS) for Mozambique informed by seven years of weekly case reports of malaria in children under 5 years of age from 142 districts. A spatio-temporal model was developed based on explanatory climatic variables to map exceedance probabilities, defined as the predictive probability that the relative risk of malaria incidence in a given district for a particular week will exceed a predefined threshold. Unlike most spatially discrete models, our approach accounts for the geographical extent of each district in the derivation of the spatial covariance structure to allow for changes in administrative boundaries over time. The MEWS can thus be used to predict areas that may experience increases in malaria transmission beyond expected levels, early enough so that prevention and response measures can be implemented prior to the onset of outbreaks. The framework we present is also applicable to other climate-sensitive diseases.

## Introduction

Malaria is a major cause of morbidity and mortality in Mozambique, with an estimated 8.3 million cases and 15,000 deaths in 2015^1^. Mozambique contributes 18% of annual malaria cases reported in the East and Southern Africa region, tied with Uganda for the highest percent contribution by any single country^2^. Malaria transmission in Mozambique is highly seasonal, and varies significantly geographically, with the highest transmission occurring in the north and the lowest in the south. Additionally, a number of unexpected increases in cases have occurred over the last several years that have resulted in significant morbidity and mortality. The country currently has an outbreak detection system called the *Canal Endemico*, which is designed to detect weekly case counts that are significantly higher than historical averages for that week. However, due to delays in reporting, outbreaks are not detected in sufficient time to mount an effective response. Thus, there is still a need to develop a system that predicts malaria epidemics with sufficient lead time to allow for effective interventions to be implemented.

Over the past two decades, researchers have developed malaria early warning systems (MEWS) to predict and detect malaria outbreaks across different parts of the world with mixed success^3^. This effort was in part inspired by a World Health Organization (WHO) Roll Back Malaria report published in 2001 detailing the need for developing epidemic warning systems for malaria in Africa^4^. The public health goal of these systems is to predict in advance areas with elevated risk for malaria transmission so that prevention and response measures can be implemented prior to the onset of outbreaks. Most MEWS employ time series models to predict levels of malaria transmission in the forthcoming weeks and months as a function of current or forecasted climatic conditions^5–8^. The practical usefulness of MEWS largely rests on the availability of good quality malaria incidence data and climatic data for developing a prediction model. Here we define climatic variables as those which affect mosquito behaviour and parasite survival at timescales from daily (e.g., daily rainfall and temperature) to weekly/monthly (e.g., heatwaves and drought/flood conditions influenced by El Niño and La Niña). Therefore, climatic variables provide useful information to model and predict seasonal fluctuations in malaria transmission risk^9–11^. In 2012, Mabaso and Ndlovu published a summary of 35 articles on malaria epidemic models, many of which reported associations between malaria incidence and temperature, rainfall and humidity^3^. Understanding of climatic influences on variation in malaria risk has been facilitated by the increasing availability of remotely sensed climatic data at high spatial resolution^12,13^.

Spatio-temporal variation in malaria transmission is ideally described through continuous monitoring of geolocated case data combined with environmental and climatic information from the location where infections occurred. However, in low-resource settings, where MEWS are most needed, cost-effective malaria surveillance systems are based on passive detection of cases reported as an aggregated count over large administrative units (e.g., county or district level) on a weekly or monthly basis. Although these data are only available at coarse spatial resolution, the association of climatic factors with malaria risk can still be established if enough geographic variation occurs at the administrative unit level, enabling prediction of future levels of transmission^7,10,14^. In addition to climatic factors, environmental and socio-economic risk factors also drive the variation in malaria risk but are often difficult to measure over a large geographic area^15^.

Girond *et al*. recently developed a Seasonal Auto-Regressive Integrated Moving Average (SARIMA) model for malaria outbreak detection in Madagascar using weekly case reports, satellite weather data and intervention data^6^. Similarly, Johansson *et al*. developed a dengue early warning system for Mexico, which included satellite weather data and monthly case reports across almost 30 years using SARIMA models, and concluded that models with short-term and seasonal autocorrelations improved prediction performance over models with only short-term autocorrelations and/or weather variables^16^. One of the main limitations of these two approaches is that the reported counts were modelled as continuous measurements and, therefore, could potentially lead to inadmissible negative estimates of incidence in the case of low counts. Additionally, these approaches do not exploit the additional benefit that might accrue from modelling the spatial, as well as temporal, correlation of the data. Indeed, almost any infectious disease, including malaria and dengue, is characterized by both spatially and temporally structured dynamics of transmission^17,18^. Mbaso *et al*.^17^ illustrated the advantage of using a spatio-temporal model to assess the effect of climate on malaria incidence in Zimbabwe using a conditional autoregressive (CAR) process. However, CAR models cannot be used when the boundaries of the administrative units change over time. Furthermore, the implied spatial correlation structure by such models between neighbouring regions is often not realistic^19^.

To facilitate decisions with financial and operational consequences, it is particularly important to convey uncertainty in estimates of malaria risk in a way that can be easily understood by public health officials. While most MEWS have an exclusive focus on the prediction of incidence^10,20,21^, in this paper we argue that the goal of a MEWS should be, instead, to quantify the probability of exceeding policy relevant thresholds which can signal the start of a major public health threat. For this reason, we also emphasize that validation of MEWS should be carried out by assessing both their accuracy in predicting incidence and their reliability in measuring uncertainty.

Risk of malaria infection in Mozambique is highly variable throughout the year and throughout the country. The rainy season begins in November or December and lasts through April or May, with the peak in malaria transmission usually occurring in February or March^1^. In order to address unpredictable patterns in transmission, we developed a MEWS for early epidemic detection in children <5 in Mozambique using weekly case reports from 142 districts from 2010 through early 2017. By defining an outbreak as the exceedance of a predefined relative risk threshold, our objective is to estimate the probability that such an event would occur in the future in any of 142 districts. By modelling the residual spatio-temporal variation in malaria incidence, we formulate a spatially discrete process whose correlation structure is derived from a spatially continuous Gaussian process. Unlike spatially discrete models based on Markov fields (e.g. CAR models), this approach allows us to account for the geographical shape and extent of each of the 142 districts. Ideally, the framework we present will allow public health officials to make more informed decisions when allocating resources that have been set aside for malaria outbreaks.

## Methods

### Data

Malaria incidence data consisted of weekly reports of confirmed cases from 142 districts in Mozambique from the first week of 2010 through the ninth week of 2017. The aggregated malaria case reports were provided by the National Malaria Control Program (NMCP) of Mozambique. In this study, we used only cases reported in children <5 years of age, as they represent the exposure group most likely to experience symptoms and therefore seek care. District population data were obtained from WorldPop^22^, and we used the 2007 Mozambique census data in order to estimate the proportion of the population <5 years of age. Climatic data were derived from the Version A forcing data of the Famine Early Warning Systems Network Land Data Assimilation System (FLDAS), available from the National Aeronautics and Space Administration Goddard Earth Science Data and Information Services Center (https://earthdata.nasa.gov/about/daacs/daac-ges-disc). The FLDAS data are available from NASA at daily temporal resolution and 0.1 degree spatial resolution. The data were aggregated to weekly temporal resolution and district-level spatial resolution by bilinearly interpolating the four nearest FLDAS grid points to the centroid of each district. Climatic variables included total weekly rainfall (mm), average weekly temperature (^o^C), average weekly relative humidity (%), average weekly surface barometric pressure (hPa) and average weekly saturation vapor pressure deficit (mmHg). These weekly, district-level climatic variables were included in the spatio-temporal model fitting process, specified in the next section, with lags at zero, two, four and eight weeks.

### Spatio-temporal modelling

We develop a spatio-temporal model for the malaria counts *Y*_*it*_ in week *t* and district *i* as follows. We assume that, conditional on spatio-temporal random effects *S*_*it*_, the *Y*_*it*_ are mutually independent Poisson variables with mean *m*_*it*_*λ*_*it*_ where *m*_*it*_ is the estimated population of children under 5 and

1 $$\mathrm{log}\{{\lambda }_{it}\}={d}_{it}^{T}\beta +{S}_{it}$$

where *d*_*it*_ is a vector of explanatory variables with associated regression coefficients *β*.

We interpret *S*_*it*_ as the residual spatio-temporal variation in log-incidence that is not explained by *d*_*it*_. At a given time *t*, we then model *S*_*it*_ as an averaged Gaussian process over district *i*, i.e.

2 $${S}_{it}=\frac{1}{|{R}_{i}|}{\int }_{{R}_{i}}S(x)dx,$$

Where *R*_*i*_ is the region encompassed by the boundaries of district *i* and $|{R}_{i}|$ is its area, and *S(x*) is a zero-mean stationary and isotropic Gaussian process with variance σ^2^ and exponential correlation function with scale parameter φ. The resulting spatial covariance functions between *S*_*it*_ and *S*_*jt*_ is then given by

3 $$Cov({S}_{it},\,{S}_{jt})=\frac{{\sigma }^{2}}{|{R}_{i}||{R}_{j}|}\iint {e}^{-\frac{\Vert x-x^{\prime} \Vert }{\phi }}dx\,dx^{\prime} .$$

Let, *S*_*t*_ = *(S*_*1,t*_*, …, S*_*142,t*_*)* be the collection of all the random effects at time *t*. The overall spatio-temporal covariance structure of the model is then defined by the following autoregressive relationship of the first order

4 $${S}_{t}=\rho {S}_{t-1}+{W}_{t},$$

where ρ is between −1 and 1, and *W*_*t*_ is a zero-mean multivariate Gaussian variable with covariance function given by Eq. (3).

Since our objective is to make predictive inferences on *λ*_*it*_, we first estimate *β* for each climatic variable (including their lags at zero, two, four and eight weeks) using a standard Poisson regression, where *S*_*it*_ = 0 for all *i* and *t*, and carry out variables selection using a LASSO penalty. We then fit the model in Eq. (2) using Bayesian inference while fixing *β* at its estimate obtained from the standard Poisson model. The reason for this is that *β*, in our case, is a nuisance parameter and, by treating this as an offset quantity in our model, we are then able to carry out the fitting more efficiently. In this modelling framework, missingness in the reported malaria cases are automatically handled within the Markov Chain Monte Carlo algorithm by simulating from the distribution of the spatio-temporal random effects of each consecutive week, conditioned on the available data. Further computational details are provided in the supplementary material.

To quantify the likelihood of occurrence of an outbreak, we use exceedance probabilities (EPs) of relative risk, given by ${e}^{{S}_{it}},$ which measures the unexpected multiplicative deviation from the expected incidence level ${e}^{{d}_{it}^{T}\beta }$. The expression for the EP is

5 $$Prob({e}^{{S}_{it}} > l|data)$$

where *l* is a predefined threshold. In the results section, we arbitrarily set *l* = *2* to compute the probability of observing an incidence level that is twice as large as what would be expected.

### Model comparison and validation

To better understand the contribution of the climate variables and the spatio-temporal random effects to our predictive inferences, we compare the “comprehensive” model in Eq. (2), that we shall denote as M3, with the two following simpler models: (M1) a naïve approach that ignores any residual spatio-temporal variation and only uses the climate variables *d*_*it*_; (M2) a model that ignores the climate variables and only uses the spatio-temporal random effects *S*_*it*_.

We assess the performance of each of the three models in terms of their ability to predict incidence and quantify the associated uncertainty for the 26 weeks (6 months) following the 35^th^ week in 2016. To this end, we then hold out the last 26 weeks of available case reports and fit the model to the remaining data. We summarize the predictive performance of the three models for each of the 26 weeks using the root-mean-square-error (RMSE), expressed as

6 $$RMS{E}_{t}=\sqrt{\frac{1}{142}{\sum }_{i=1}^{142}\,{({\lambda }_{it}^{emp}-{\hat{\lambda }}_{it})}^{2}}\,$$

Where $\,{\lambda }_{it}^{emp}\,$is the empirical incidence computed from the raw data and ${\hat{\lambda }}_{it}$ is the posterior mean of incidence from the fitted model; to assess the reliability of a model to measure uncertainty, we use the 95% coverage probability (CP), formally defined as

7 $$C{P}_{t}=\frac{1}{142}\sum _{i=1}^{142}\,I({\lambda }_{it}^{0.025} < {\lambda }_{it}^{emp} < {\lambda }_{it}^{0.975}).$$

where $I({\lambda }_{it}^{0.025} < {\lambda }_{it}^{emp} < {\lambda }_{it}^{0.975})$ is an indicator function that takes value 1 if ${\lambda }_{it}^{0.025} < {\lambda }_{it}^{emp} < {\lambda }_{it}^{0.975}$ and 0 otherwise, with ${\lambda }_{it}^{0.025}$ and ${\lambda }_{it}^{0.975}$ corresponding to the quantiles 0.025 and 0.975 of the posterior distribution for ${\lambda }_{it}$, respectively.

## Results

**Figure 1:**
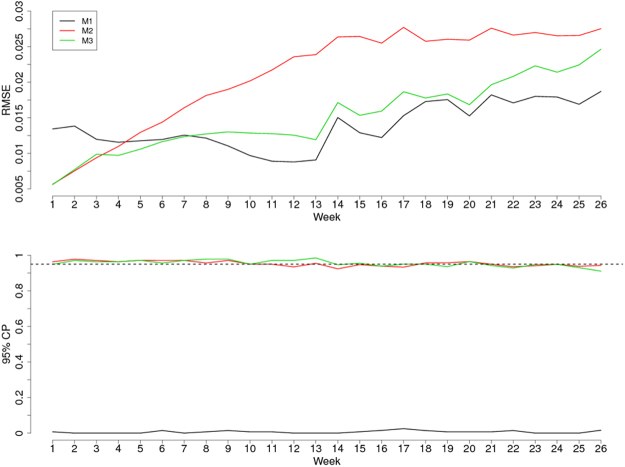
Statistics comparing model performance. Root-mean-square-error (RMSE) and 95% coverage probabilities (CP) for: the model that only uses the climate data (M1); the model without the climate data and purely based on the spatio-temporal random effects; the model that combines both the climate data and spatio-temporal random effects (M3). The horizontal dashed line in the lower panel corresponds to 0.95 which is the nominal level of coverage for the prediction intervals.

The upper panel of Fig. 1 shows the root-mean-square-error (RMSE) by week for the three models using a hold-out sample consisting of the last 26 weeks of reported cases. In the first three weeks, M2 (random effects only) has the lowest RMSE, while from the fourth to the seventh week M3 (comprehensive) has the lowest RMSE. From the eighth week onwards, M1 (climate only) has the lowest RMSE. This indicates that the residual spatio-temporal correlation in M2 and M3 that we model through the random effects *S*_*it*_ enables more accurate predictions in the short-term. However, M2 does not account for any seasonal variation and this is also evident by the fact that from the fifth week onward this model yields the highest values in RMSE.

In the lower panel of Fig. 1, we report the 95% CP for the 26 weeks of the hold-out samples. M1 is by far the worst performing model with values of CP close to zero throughout the 26 weeks. This indicates that M1 severely underestimates variance, which is not surprising since M1 ignores the extra-Poisson variation induced by the residual spatio-temporal correlation and, therefore, provides unreliable measures of uncertainty. M2 and M3, on the other hand, show CP values close to 95% for all the 26 weeks.

**Figure 2:**
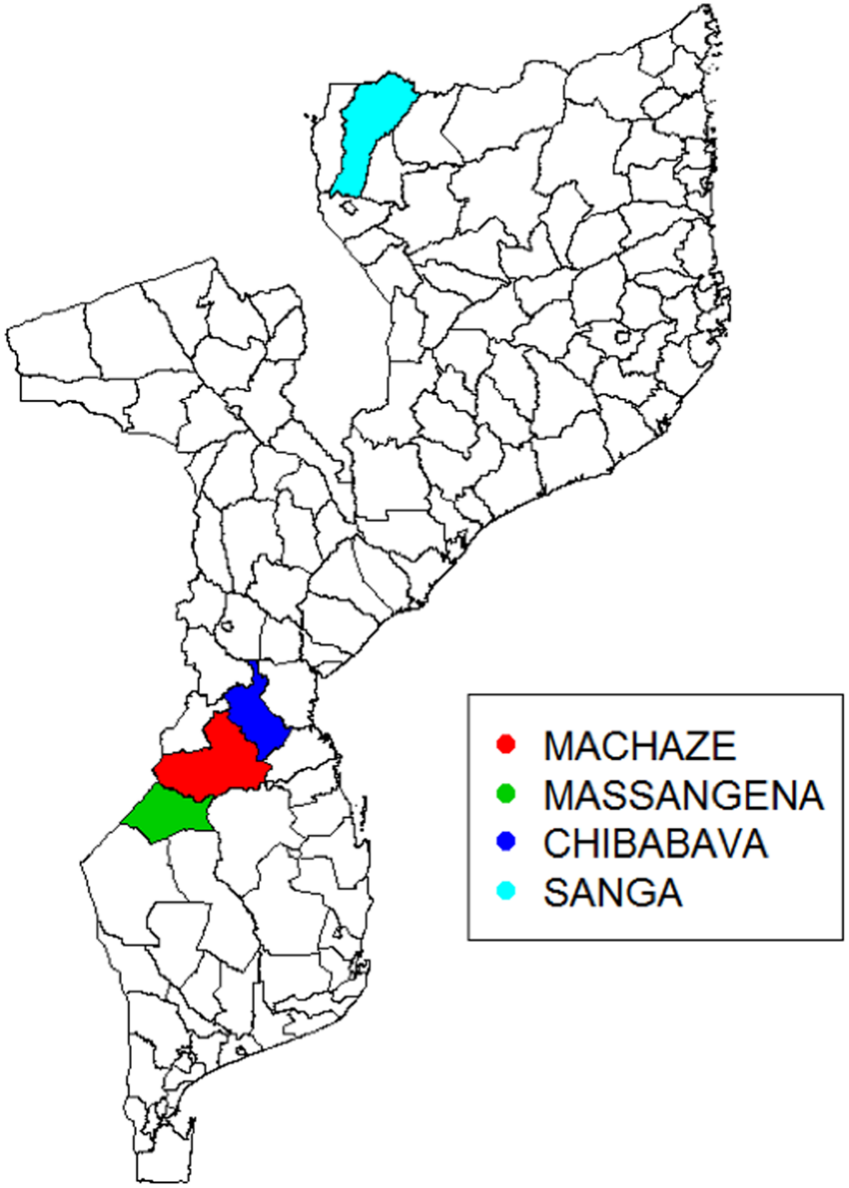
Map of Mozambique’s administrative districts. Map of the administrative districts of Mozambique with four highlighted districts as indicated by the legend.

In light of the results from Fig. 1, we chose M3 as the best model because it produced low RMSE and accurately estimated the variance. We then used M3 to carry out the spatio-temporal predictions for incidence and compute the exceedance probabilities (EPs) of a relative risk threshold of 2; i.e. the probability of observing levels of malaria incidence that would be twice larger than expected. To better understand the usefulness of using EPs, we computed them using two sets of hold-out samples, a first set consisting of 26 weeks from the 35th week in 2016 to the 9th week in 2017, and a second set of 8 weeks spanning from the 2nd to the 9th week of 2017. Malaria outbreaks were indeed observed in early 2017 in southeastern and northwestern Gaza, western Manica, Sofala, and western Niassa Provinces. For this reason, we then show our results by focusing on four districts where the outbreak occurred (shown in Fig. 2): Machaze (Manica Province), Massangena (Gaza Province), Chibabava (Sofala Province) and Sanga (Niassa Province).

**Figure 3:**
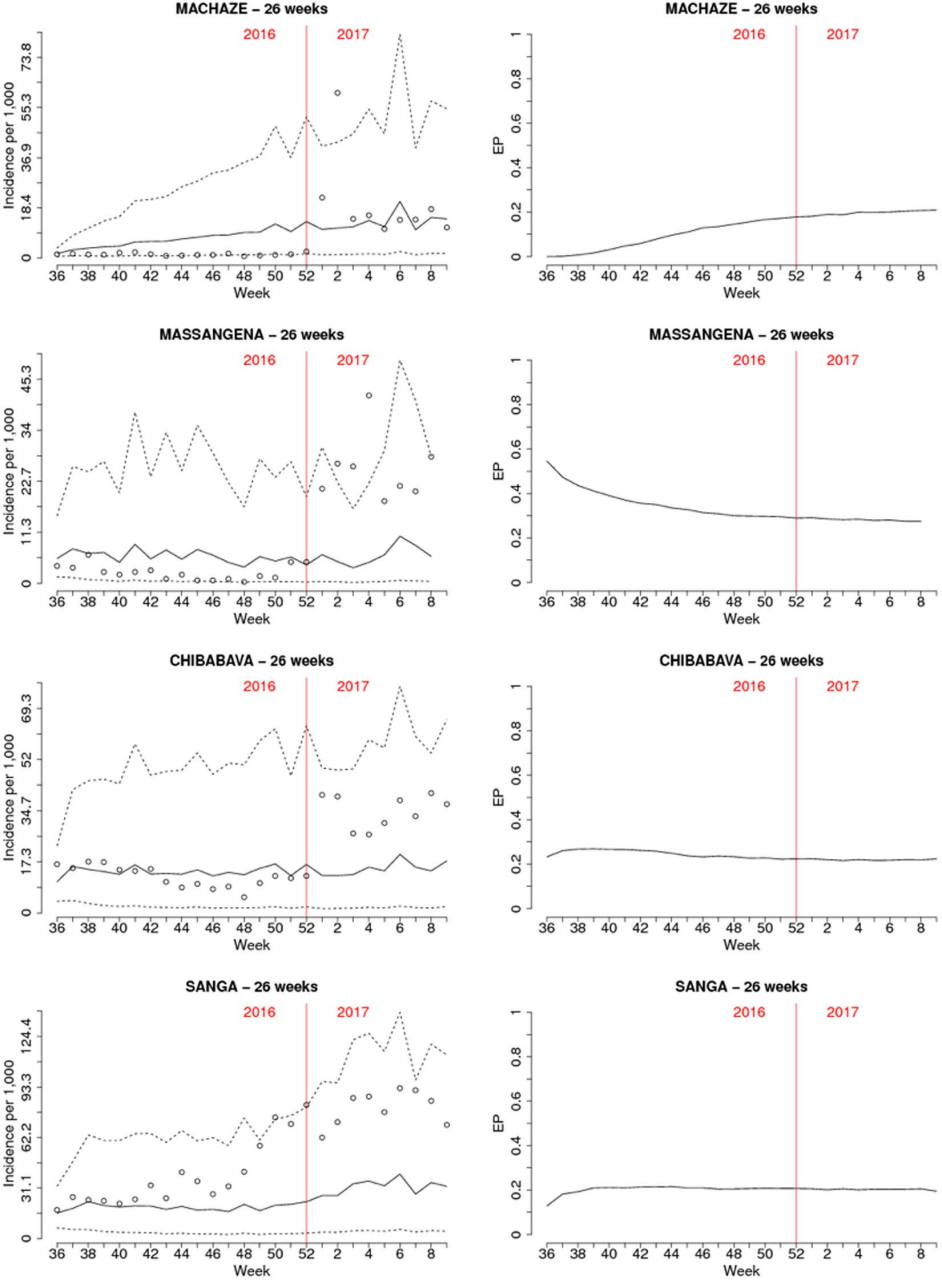
Prediction results for the districts of Machaze, Massangena, Chibabava, and Sanga over the following 26 weeks after the 35^th^ week in 2016. The left panels show the posterior mean (solid line) and 95% credible intervals (dashed lines) for malaria incidence. The right panels show the exceedance probability for a relative risk threshold of 2. Dots show observed incidence. The results for the 9th week in 2017 of Massangena district are not reported because the incidence data were not available.

**Figure 4:**
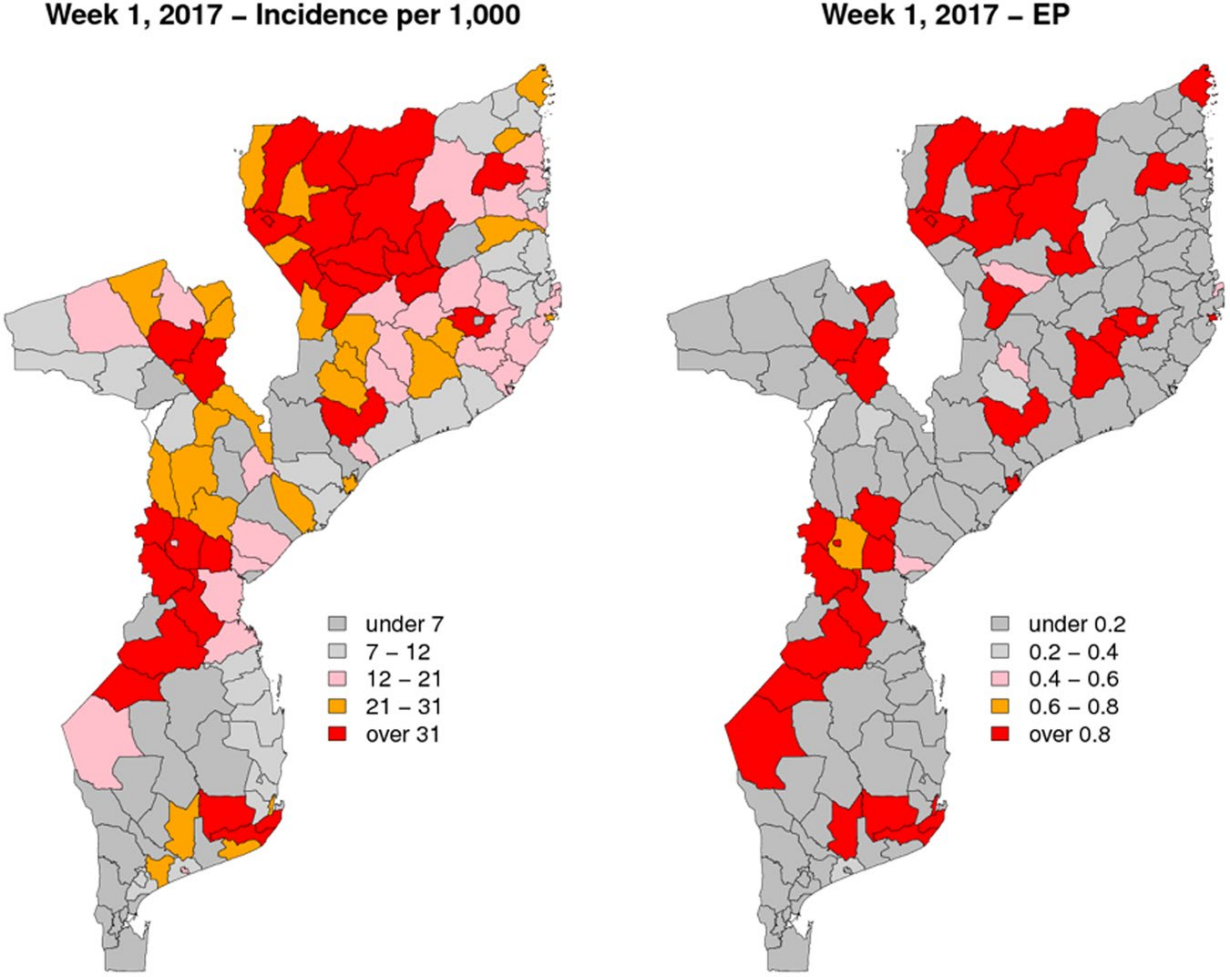
Prediction results for the 1^st^ week of 2017. The left panel shows the posterior mean of malaria incidence for each of the 142 districts. The right panel shows the exceedance probability for a relative risk threshold of 2.

**Figure 5:**
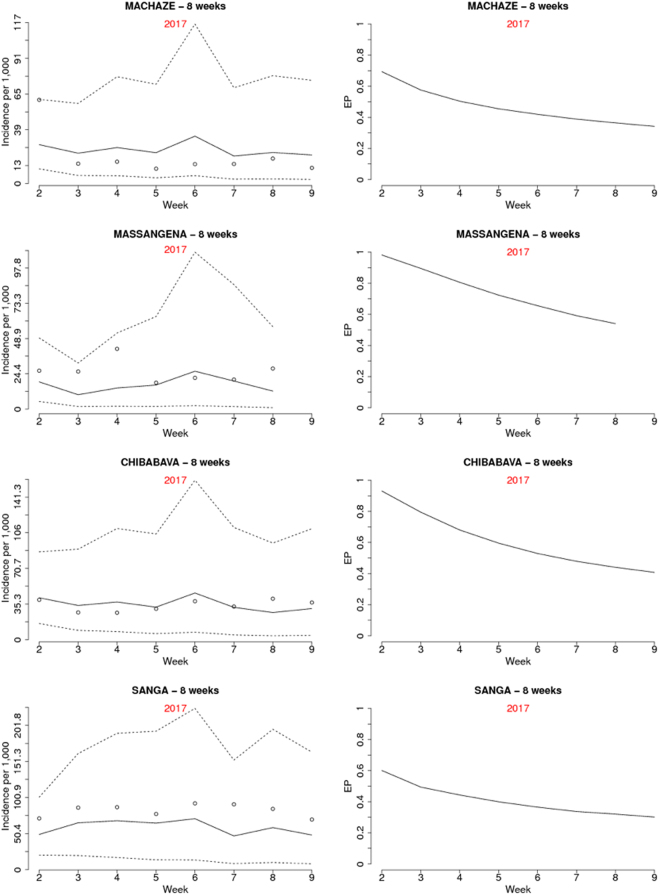
Prediction results for the districts of Machaze, Massangena, Chibabava, and Sanga over the following 8 weeks after the 1^st^ week in 2017. The left panels show the posterior mean (solid line) and 95% credible intervals (dashed lines) for malaria incidence. The right panels show the exceedance probability for a relative risk threshold of 2. Dots show observed incidence. The results for the 26th week (the 8th week of the hold-out sample) of Massangena district are not reported because the incidence data were not available.

Figure 3 shows the predicted incidence with associated 95% prediction intervals (left panels) and the EPs (right panel) for the four districts over the 26 weeks of the first set of hold-out samples. While the 95% prediction intervals provide information about the uncertainty around estimates of incidence, the EPs are threshold-specific and are exclusively focused on the unexpected variation in incidence. The EPs reach their highest value of about 45% in Massangena but never become high enough to signal the start of a potential outbreak over the 26 weeks (one would ideally set the threshold well above 50%, for example, at 80% or greater). This is because EPs are informed by the spatio-temporal random effects *S*_*it*_ for which the data provide stronger information for weeks that are closer to the last week of reported cases. This is also evident when carrying out the predictions for the 8 week period of the second hold-out sample, where EPs now signal the high likelihood of outbreaks (Figs 4 and 5). Figure 4 shows the estimated malaria incidence (left panel) and EPs (right panel) for the last week of the training sample, which correspond to the first week of 2017. The estimated incidence shows values above 31 per 1,000 in several districts across the country for the first week of January 2017, and most of these are found in the north at the boundary with Tanzania. In the map of the EPs, districts that are coloured in red are associated with an EP of at least 80%, thus indicating a high likelihood that incidence will be at least twice what would be expected. Over the 8 weeks that follow the first week in 2017 (weeks 2 through 9), the interpolated malaria incidence (Fig. 5, left panel), albeit high in value, does not convey any indication whether these estimated levels of transmission are indeed the manifestation of an outbreak. This is because the solid lines in the left panels of Fig. 5 are the product of two components: the cumulative effects from weather variables and the spatio-temporal random effects. However, based on our definition of an outbreak, our interest lies in the second component of incidence, i.e. the unexplained variation. The EPs (Fig. 5, right panel) are very close to 100% in Massangena and Chibabava for up to two weeks ahead, meaning that incidence was highly likely to have exceeded expected levels by at least a factor of 2. In Machaze, the EP is close to 80% in the first week and decreasing afterwards, reaching about 20% in the eighth week. In the case of Sanga, there is a higher level of uncertainty on its exceedance, or not, of a threshold of 2 in relative risk: the first week shows an EP of about 50%, the highest possible value of uncertainty, which then decreases to about 20% in the eighth week.

If an outbreak were to be declared whenever an EP is no less than 75%, this would occur in Massangena from weeks 2 to 4 and in Chibabava from weeks 2 to 3 (Fig. 5). If instead a stricter rule for signalling the onset of an outbreak is used, e.g. an EP no less than 90%, this would occur in Massangena and Chibabava in week 2 only.

The decreasing trend in EPs for all four districts is due to the fact that as we move further in time from the last week of reported cases, our best guess for the unexplained variation *S*_*it*_ is that this will revert toward its prior mean and, therefore, ${e}^{{S}_{it}}$ is less likely to exceed 2. The posterior mean for the coefficient of temporal correlation ρ is about 0.86, thus leading to a correlation between spatio-temporal random effects associated with the same district of about 0.56 for 4 weeks apart and 0.31 for 8 weeks apart.

## Discussion

The ability to accurately and confidently predict malaria outbreaks is a necessary component of a highly functional malaria control/elimination program. MEWS are rarely used in practice in Africa, largely because most lack either the accuracy or precision to be adopted by malaria control programs or because of constraints on resources and logistics. Predicting too many outbreaks leads to the exhaustion of resources and eventually the inability to mount any effective response, while predicting too few outbreaks erodes confidence in the system and eventual lack of use. In this study, we developed a MEWS based on a novel spatio-temporal model to generate EPs for the unexpected variation in malaria incidence. Rather than providing a binary yes/no classification that an outbreak will occur, the use of EPs provides the likelihood that malaria incidence in a given week will be higher than expected. This system therefore allows the end-user to set levels of certainty for outbreak detection that are appropriate for the level of resources available in the country. For example, the end-users may start with an EP threshold of 70%, and if that yields too many districts to which they need to respond given their resources, then they may choose to increase the EP threshold to 80%. Alternatively, the end-users could set an EP threshold at 75%, then further reduce the number of districts to which they target resources based on the potential number of infections that might be prevented (i.e., prioritize districts with larger populations).

The ability of the developed MEWS to signal the onset of potential outbreaks rests in the accuracy of our predictions for the spatio-temporal random effects *S*_*it*_, as a large uncertainty in these would lead to EPs closer to 50%. Our results indicate that the correlation in time between the *S*_*it*_ at the same district is no less than 0.55 if we consider two time points 4 weeks apart. This also suggests that the data might be informative with respect to outbreaks occurring within a 4 weeks time window but increasingly less informative for later weeks.

In addition to measures of incidence, a MEWS needs to take into account unmeasured factors related to malaria risk. The model we developed for this MEWS does this through the spatio-temporal random effects. Furthermore, unlike other spatially discrete models based on Markov structures (e.g. CAR models) that are tied to an arbitrary partition of study area, our modelling approach allowed us to account for the geographical size of each of the 142 districts in the derivations of the covariance structure and to overcome the potential issue of changes in the boundaries of administrative areas.

Climatic drivers play a clear role in the dynamics of malaria transmission, though the extent and nature of this role is highly variable geographically^9–11^. Although this study was not designed to identify which specific climatic variables were most associated with malaria transmission, the fact that the inclusion of these variables improved the prediction performance of the model (Fig. 1) is consistent with what has been reported in previous studies^3,9–11^. Moreover, our approach of carrying out variables selection using a LASSO penalty allowed for only those climatic variables that were important for prediction to be included. We found that the specific variables selected depended on the week one wanted to predict; therefore, the model is adaptive over time (i.e., for some weeks all variables were selected and for other weeks only a few were selected). We recognize there is a potential limitation of using aggregated weather data over an entire district, but in our view, the programmatic advantages of using district-level incidence estimates outweigh these limitations. Furthermore, local knowledge of transmission foci within districts may enable interventions to be targeted at specific areas within districts predicted as having elevated risk.

We did not include variables that relate to control interventions in these models. The two major vector control interventions that have been implemented over the past decade in Mozambique are distribution of insecticide treated bednets (ITN) and indoor residual spraying of households (IRS). The NMCP has a detailed record of the location and timing of interventions over the past decade; however, inclusion of these data into a MEWS is not trivial. ITN campaigns are done at the district level, but national distribution of bednets began in 2012, concurrent with an increase in reported cases, so the associations between ITN distribution and malaria incidence in a model might appear to be erroneously positive. Furthermore, IRS campaigns are typically applied at the district level, but they often take several months to complete, and therefore it is difficult to determine when coverage began and ended for each district. Moreover, the districts with the highest historic incidence typically receive IRS, so it again would appear to be associated with higher incidence.

We also recommend periodic re-validation of the model as malaria transmission within a region changes. For example, if a region of the country was approaching elimination, it might be reasonable to pursue a different approach, such as case-based mapping and response, as described by Cohen *et al*.^23^.

We are currently developing a web application with a friendly user interface for visualization of the model outputs that will ideally be used as a decision-support tool by the NMCP in Mozambique. Furthermore, the MEWS framework we have developed could also be used to monitor other diseases in low-resource settings with spatio-temporal dynamics of transmission, such as dengue, Zika virus and cholera.

### Data availability

The malaria incidence data are the property of the NMCP of Mozambique and can be made available on reasonable request. The weather data can be freely accessed through the FLDAS (https://ldas.gsfc.nasa.gov/FLDAS/).

## Electronic supplementary material

Supplementary Information

## References

[1] WHO. World Health Organization: Mozambique. *Geneva: World Health Organization* (2017).

[2] WHO. Fact Sheet: World Malaria Report 2016. *Geneva: World Health Organization* (2016).

[3] Mabaso ML, Ndlovu NC (2012). Critical review of research literature on climate-driven malaria epidemics in sub-Saharan Africa. Public Health.

[4] WHO. Roll Back Malaria. Malaria early warning system. A framework for Field Research in Africa: concepts, indicators and partners. *Geneva: World Health Organization* (2001).

[5] Abeku TA, van Oortmarssen GJ, Borsboom G, de Vlas SJ, Habbema JD (2003). Spatial and temporal variations of malaria epidemic risk in Ethiopia: factors involved and implications. Acta Trop.

[6] Girond F (2017). Analysing trends and forecasting malaria epidemics in Madagascar using a sentinel surveillance network: a web-based application. Malaria journal.

[7] Merkord CL (2017). Integrating malaria surveillance with climate data for outbreak detection and forecasting: the EPIDEMIA system. Malaria journal.

[8] Midekisa A, Senay G, Henebry GM, Semuniguse P, Wimberly MC (2012). Remote sensing-based time series models for malaria early warning in the highlands of Ethiopia. Malaria journal.

[9] Bouma MJ, van der Kaay HJ (1996). The El Nino Southern Oscillation and the historic malaria epidemics on the Indian subcontinent and Sri Lanka: an early warning system for future epidemics?. Trop Med Int Health.

[10] Thomson MC (2006). Malaria early warnings based on seasonal climate forecasts from multi-model ensembles. Nature.

[11] Wu Y (2017). Describing interaction effect between lagged rainfalls on malaria: an epidemiological study in south-west China. Malaria journal.

[12] Midekisa A, Beyene B, Mihretie A, Bayabil E, Wimberly MC (2015). Seasonal associations of climatic drivers and malaria in the highlands of Ethiopia. Parasites & vectors.

[13] Sewe MO, Tozan Y, Ahlm C, Rocklov J (2017). Using remote sensing environmental data to forecast malaria incidence at a rural district hospital in Western Kenya. Scientific reports.

[14] Thomson MC, Connor SJ, Milligan P, Flasse SP (1997). Mapping malaria risk in Africa: What can satellite data contribute?. Parasitol Today.

[15] Giardina F (2014). Effects of vector-control interventions on changes in risk of malaria parasitaemia in sub-Saharan Africa: a spatial and temporal analysis. The Lancet. Global health.

[16] Johansson MA, Reich NG, Hota A, Brownstein JS, Santillana M (2016). Evaluating the performance of infectious disease forecasts: A comparison of climate-driven and seasonal dengue forecasts for Mexico. Scientific reports.

[17] Mabaso ML, Vounatsou P, Midzi S, Da Silva J, Smith T (2006). Spatio-temporal analysis of the role of climate in inter-annual variation of malaria incidence in Zimbabwe. Int J Health Geogr.

[18] Martinez-Bello DA, Lopez-Quilez A, Torres Prieto A (2017). Relative risk estimation of dengue disease at small spatial scale. Int J Health Geogr.

[19] Wall MM (2004). A close look at the spatial structure implied by the CAR and SAR models. Journal of Statistical Planning and Inference.

[20] Hay SI (2003). Forecasting, warning, and detection of malaria epidemics: a case study. Lancet.

[21] Teklehaimanot HD, Lipsitch M, Teklehaimanot A, Schwartz J (2004). Weather-based prediction of Plasmodium falciparum malaria in epidemic-prone regions of Ethiopia I. Patterns of lagged weather effects reflect biological mechanisms. Malaria journal.

[22] WorldPop. WorldPop: detailed and open access population distribution datasets built using transparent approaches. *WorldPop* (2017).

[23] Cohen JM (2013). Rapid case-based mapping of seasonal malaria transmission risk for strategic elimination planning in Swaziland. Malaria journal.

