# Supplementary material for: Spatio-temporal modelling of weekly malaria incidence in children under 5 for early epidemic detection in Mozambique
Source: Sci Rep. 2018 Jun 18;8:9238. doi: 10.1038/s41598-018-27537-4 (PMC6006329; doi:10.1038/s41598-018-27537-4)
Supplement: Supplementary file 1 — Supplementary Information [file 41598_2018_27537_MOESM1_ESM.pdf]

Spatio-temporal modelling of weekly malaria incidence in children under 5 for early epidemic detection in Mozambique

Kathryn L. Colborn[1*], Emanuele Giorgi[2], Andrew J. Monaghan[3], Eduardo Gudo[4], Baltazar Candrihno[5], Tatiana J. Marrufo[4], James M. Colborn[6]

[1]Department of Biostatistics and Informatics, University of Colorado Anschutz Medical Campus, Aurora, CO, USA,

[2] Lancaster Medical School, Lancaster University, UK,

[3]National Center for Atmospheric Research, Boulder, CO, USA,

[4]Instituto Nacional de Saude, Maputo, Mozambique,

[5]National Malaria Control Program, Maputo, Mozambique,

[6]Clinton Health Access Initiative, Boston, MA, USA

[*]Corresponding author: Kathryn Colborn, 13001 E. 17th Place, Room C3011, University of Colorado Anschutz Medical Campus, Aurora, CO 80045; email: Kathryn.Colborn@ucdenver.edu; phone: 303-946-3578

1 Model formulation

Let Y_{it} be random variable of the number of reported malaria cases for children under 5 years in district $\mathcal{R}_i$ and epidemiological week t. We assume that conditionally on a Gaussian spatio-temporal random effect S_{it}, the Y_{it} are mutually independent Poisson variables with mean

$$\lambda_{it} = m_{it} \exp\{d_{it}^\top \beta + S_{it}\}$$

where d_{it} is a vector of spatio-temporal explanatory variables with associated regression coefficients β.

Let $S_t^\top = (S_{1t}, \dots, S_{nt})$ denote the collection of the random effects associated to each of the $n = 142$ districts in Mozambique in week t. We then assume S_t to follow a multivariate Gaussian distribution with mean zero and covariance matrix Σ such that

$$[\Sigma]_{ij} = \frac{\sigma^2}{|\mathcal{R}_i||\mathcal{R}_j|} \int_{\mathcal{R}_i} \int_{\mathcal{R}_i} \exp\{-\|x_i - x_j\|/\phi\}\, dx_i dx_j$$

where $|\mathcal{R}_j|$ is the area encompassed by the boundaries of district $\mathcal{R}_i$. We approximate the above integral using a numerical integration procedure based on a 1 km^2 regular grid covering the whole of Mozambique, which yields

$$[\Sigma]_{ij} \approx \frac{\sigma^2}{N_i N_j} \sum_{\tilde{x}_i \in \mathcal{R}_i} \sum_{\tilde{x}_j \in \mathcal{R}_i} \exp\{-\|\tilde{x}_i - \tilde{x}_j\|/\phi\}, \tag{1}$$

where $\tilde{x}$ are the points on the grid and N_i are the number of points $\tilde{x}$ falling within $\mathcal{R}_i$.

We model the temporal correlation between the $Y_i t$ using an autoregressive process of the first order, i.e.

$$S_t = \rho S_{t-1} + W_t, \quad -1 < \rho < 1$$

where W_t is the temporal innovation following a multivariate Gaussian distribution with mean zero and covariance matrix give by (1).

From the above assumptions, it follows that the log-density of the joint distribution of the S_t is given by

$$\log f(S_1, \dots, S_T) = \log f(S_1) + \sum_{t=2}^{T} f(S_t|S_{t-1}), \tag{2}$$

where

$$\log f(S_1) = -\frac{1}{2}\left[n \log\{2\pi\} + \det\{(1-\rho^2)^{-1}\Sigma\} + (1-\rho^2) S_1^\top \Sigma^{-1} S_1\right]$$

and

$$\log f(S_t|S_{t-1}) = -\frac{1}{2}\left[n \log\{2\pi\} + \det\{\Sigma\} + (S_t - \rho S_{t-1})^\top \Sigma^{-1} (S_t - \rho S_{t-1})\right], \quad t = 1, \dots, T.$$

2 Priors specification

We assume the following set of independent priors:

- σ^2 follows a Gaussian distribution left truncated in 0, with mean 1 and variance 10^4;
- ρ is uniform in the interval $(-1, 1)$;
- finally, we use a uniform prior for ϕ over the discrete set

$$\phi_i = 17 + i \times \frac{5}{19}, \quad i = 0, \dots, 19. \tag{3}$$

The use of a discrete prior for ϕ allows us to make the implementation of the Monte Carlo Markov chain (MCMC) faster by pre-computing the covariance matrix in (1) for all the pre-defined values ϕ_i.

We remind that, in fitting the model, the regression coefficients β are fixed at their maximum likelihood estimate from a standard Poisson model.

3 Implementation of the Markov chain Monte Carlo algorithm for Bayesian inference

We developed an MCMC to simulate from the posterior distributions of σ^2, ρ, ϕ and $(S_1, \ldots, S_T)$ by updating each of these in turn as follows.

1. ϕ: we use a Metropolis Hastings (MH) procedure and propose a new value ϕ_{prop} given the current value ϕ_{curr} based on a discretized Gaussian distribution with mean ϕ_{curr} and variance 4.

2. σ^2: we use an MH procedure and propose a new value σ^2_{prop} on the log-scale by simulating from a Guassian distribution with mean $\log\{\sigma^2_{curr}\}$ with variance h which is adaptively tuned in order to obtain a acceptance rate of 23.4%.

3. ρ: we use an MH procedure on the transformed scale $\log\{(1 + \rho)/(1 - \rho)\}$ using a Gaussian proposal with mean $\log\{(1 + \rho_{curr})/(1 - \rho_{curr})\}$ and variance h which we tune in the same way as for σ^2.

4. $(S_1, \ldots, S_t)$: we use a Hamiltonian Monte Carlo algorithm based on the leapfrog method (Neal, 2011, pages 121-122) by generating the number of steps and their size from two uniform distributions within the intervals $(0.0001, 0.15)$ and $(2, 51)$, respectively.

We run the MCMC for 110,000 iterations with a burnin of 10,000 samples and retaining every tenth sample to obtain a final set of 10,000 samples.

Figures 1 and 2 show trace plots and correlograms of the posterior samples form the MCMC for the best model M3, defined in the main manuscript, which is based on a hold-out sample of 26 weeks. The results show a good mixing of the samples, thus indicating convergence of the MCMC.

References

NEAL, R. M. (2011). MCMC using Hamiltonian dynamics. In *Handbook of Markov Chain Monte Carlo*, S. Brooks, A. Gelman, G. Jones & X.-L. Meng, eds., chap. 5. Chapman & Hall, CRC Press, pp. 113–162.

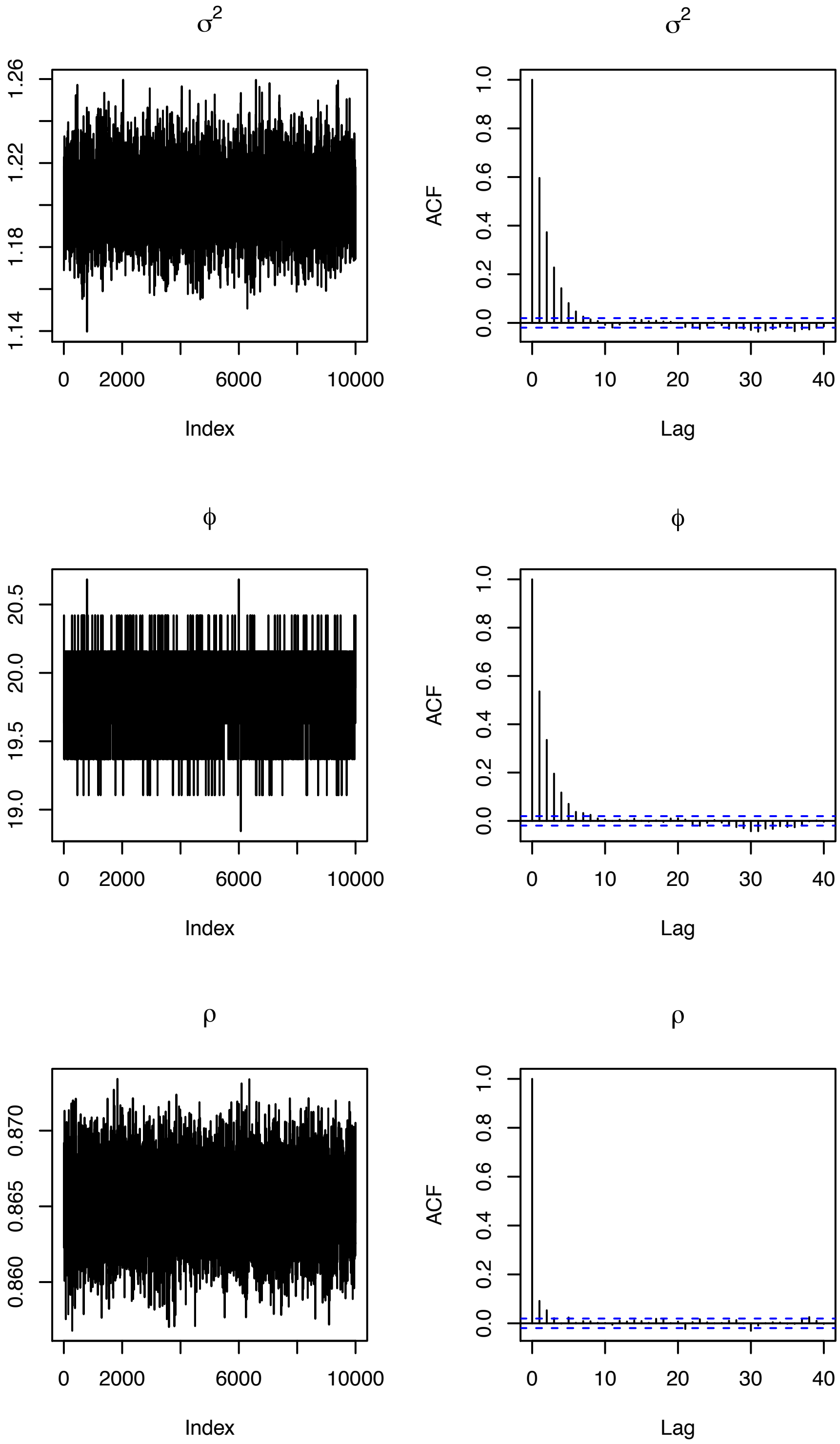

Figure 1: Trace plots (left panels) and correlograms (right panels) of the posterior samples for σ^2 (upper panels), ϕ (central panels) and ρ (lower panels).

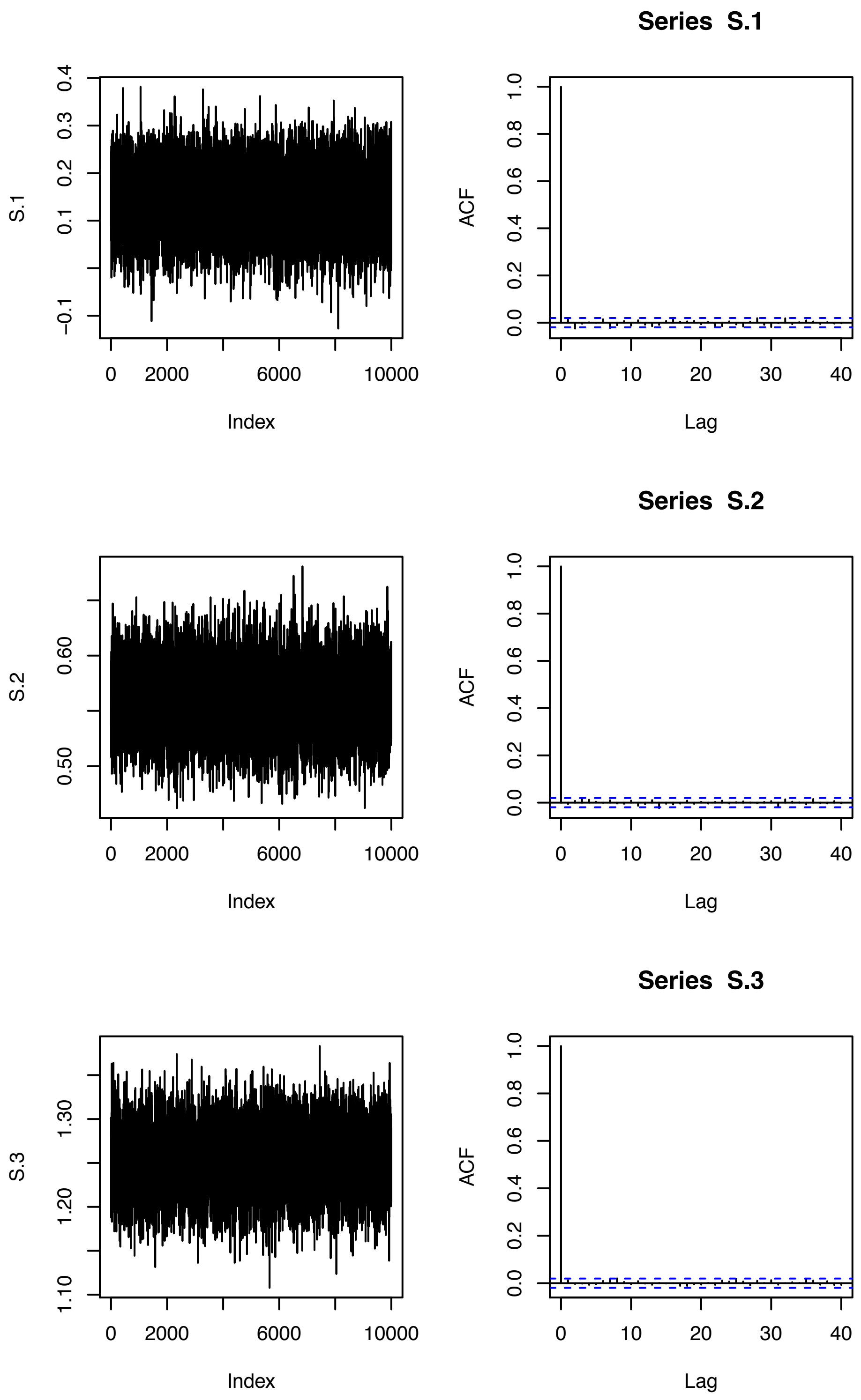

Figure 2: Trace plots and correlograms of three randomly selected components of the spatio-temporal random effects S_{it}.

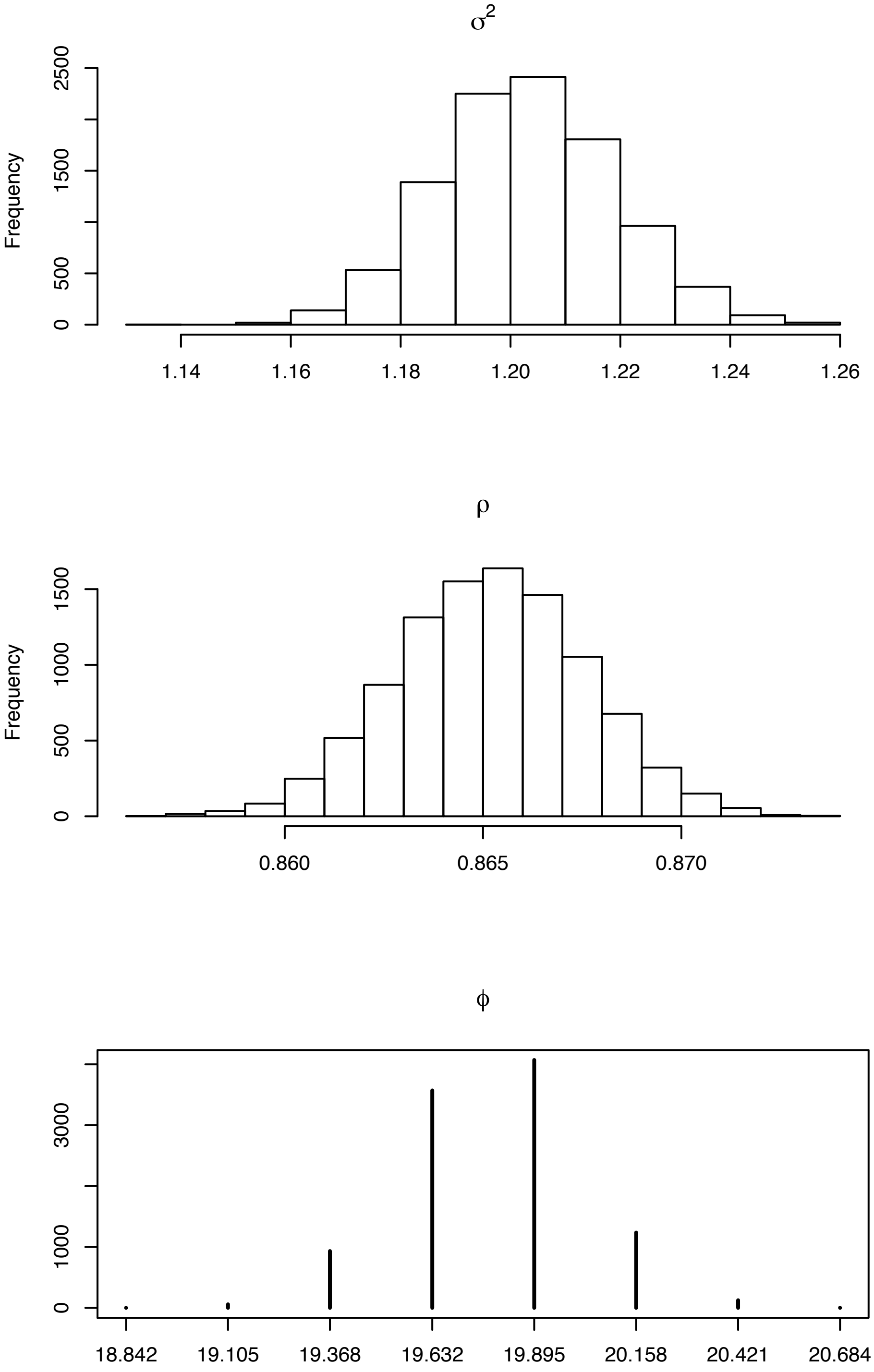

Figure 3: Histograms of the poster samples for σ^2, ρ and ϕ. The discrete posterior distribution of ϕ is visualized as a bar plot.
